# Regulatory Futures and Medical Devices: Where Next for Europe and the United Kingdom?

**DOI:** 10.5204/lthj.3174

**Published:** 2023-11-21

**Authors:** Muireann Quigley, Laura Downey, Joseph Roberts

**Affiliations:** University of Birmingham, United Kingdom

The symposium in this issue of *Law, Technology and Humans* brings together a range of scholars looking at the broad question of where next for medical devices regulation in the European Union (EU) and the United Kingdom (UK). Initially arising out of a workshop held in September 2022,^1^ our motivation for the symposium is rooted in the challenges raised by what has been a significant period of change in both the EU and the UK when it comes to medical devices regulation.

In the EU, although delayed because of the COVID-19 pandemic, Member States saw the full implementation of two new medical devices regulations: Medical Devices Regulation 2017/865 (EU MDR) and In Vitro Diagnostic Regulation 2017/866 (EU IVDR).^2^ Alongside this, the EU has also introduced, or is in the process of introducing, other legislation that will sit alongside the MDR and IVDR and which will all affect the medical devices landscape. These include: (1) the *Data Act*, which is expected to apply to all sectors using data;^3^ (2) a new legal framework on artificial intelligence (AI), including AI as a medical device (AIaMD);^4^ and (3) cybersecurity measures, including the *Cybersecurity Act*^5^ and a revised Directive on Security of Network and Information Systems (NIS2 Directive).^6^

Meanwhile, the UK has been through a protracted EU exit process that has, amongst other things, resulted in a dual system of regulation between Northern Ireland (NI) and Great Britain (GB - England, Wales, and Scotland). Although both NI and GB are directly governed by the UK’s Medical Devices Regulations 2002 (SI 2002/618, as amended), amendments as part of the Brexit process mean that NI is now subject to the requirements of the newer EU regulations, while GB continues to be governed by pre-Brexit law derived from older EU directives. One result of all this is that there has been a ‘layering of regulation upon regulation, leaving [the UK] with a fragmented, complex, and unwieldy corpus of law relating to medical devices.’^7^ The provisions of the UK’s new *Medicines and Medical Devices Act 2021* will likely exacerbate all of this. This Act, introduced and passed at the same time that some of the Brexit changes were going through Parliament, contains a wide-ranging set of powers enabling the Secretary of State to both amend existing regulations and create new ones. The introduction of new regulations as a consequence of these enabling powers is imminent. Although, at the time of writing, we do not know the exact shape or timing of these regulations, in light of the recent consultation by the UK regulator—the Medicines and Healthcare products Regulatory Agency (MHRA) on the future of medical devices regulation in the UK^8^— and the government response to these,^9^ it seems certain that they are on the horizon.

Each of the seven articles in this symposium tackles some of the gaps and challenges currently being faced regarding the regulation of medical devices in the EU and the UK. These contributions range from questions of what to do when medical devices go wrong (Macleod) and the limitations of current law and regulation (Quigley and colleagues) to how best to address cybersecurity concerns relating to medical devices (Biasin and colleagues; Ludvigsen). Three of the contributions also deal with pressing questions relating to regulating emerging (medical device) technologies (McMillan; Li; Cronin).

## (Failures of) Medical Devices Regulation

In the first contribution in this collection, Sonia Macleod describes the findings of the recent Independent Medicines and Medical Devices Safety Review (also known as the Cumberlege Review) in the UK.^10^ Set up in response to patient concerns surrounding three medical interventions (hormone pregnancy tests, valproate use in pregnancy and pelvic mesh implants), the review made recommendations aimed at improving patient safety in the UK, including, amongst others: the issuing of an apology, the establishment of a patient safety commissioner, the creation of an implantable device database, and the creation of a redress scheme for patients who are harmed by medical devices. In her article, Dr Macleod examines the circumstances that led to the Review, outlines its findings and recommendations, and evaluates the progress made so far in implementing these recommendations. She clearly demonstrates how the voices of those most affected were ignored and minimised within a system replete with multiple failures. She argues that although significant improvements have been made, risks remain. She argues that there is still scope for further improving patient safety regarding medical devices, be that via regulation or other measures.

Continuing with a focus on the UK, Muireann Quigley, Laura Downey, Zaina Mahmoud, and Jean McHale explore the complex and still-changing post-Brexit regulatory landscape for medical devices.^11^ They demonstrate how secondary legislation amending the EU-derived Medical Devices Regulations 2002 has created a dual system of regulation between NI and GB that potentially privileges NI with respect to the rest of the UK. They also show how the proliferation of secondary legislation relating to medical devices has created voluminous and, arguably, opaque law. This complexity has been exacerbated by the passing of the Medicines and Medical Devices Act 2021. Quigley and colleagues argue that the wide-ranging delegated powers to amend the medical device regulations contained in the Act allow a critical area of public policy to be governed via secondary legislation, running the risk of future inadequate scrutiny. Finally, they examine the MHRA’s 2022 consultation on the future of medical device regulation in the UK. They show that although some of the proposals diverge from the new EU regulations, aiming instead for international alignment, many of the other provisions are strikingly similar. How successful these changes will be remains to be seen, given the challenges of life outside the EU.

## Cybersecurity and Medical Devices

Two of the papers in this symposium focus on the cybersecurity challenges posed by software-enabled attached and implanted medical devices. In their contribution, Elisabetta Biasin, Erik Kamenjašević, and Burcu Yaşar examine recent changes to the EU framework for medical device cybersecurity, focusing in particular on the *Artificial Intelligence Act*, the European Health Data Space Regulation (EHDS), and the Data Act.^12^ They argue that, although these regulations make substantive changes, the fact that they are not well integrated with existing cybersecurity laws means that they may be inadequate as a means of ensuring the cybersecurity of medical devices. To remedy these shortcomings, Biasin and colleagues propose that EU legislators should define both ‘cybersecurity’ and ‘major cybersecurity incident’ more clearly. They make the persuasive case that clarity is needed regarding whether medical devices themselves count as electronic health records and thus fall under the EHDS. They also demonstrate the need for considerably stronger security requirements with respect to secure data processing environments.

The second paper examining medical devices’ cybersecurity is courtesy of Kaspar Rosager Ludvigsen.^13^ He compares how cybersecurity in relation to medical devices is currently dealt with by the law in the EU, the United States and GB and makes the case that we need a wider systems approach in this area. Ludvigsen presents and defends a novel model for thinking about how cybersecurity influences different parts of the medical device ecosystem. He sets out three domains of concern (to be thought of as ‘nested layers’), all of which need to be adequate in terms of cybersecurity for the system as a whole to function as it ought. He makes a compelling case that reform is needed to achieve this and, to this end, proposes four recommendations for law- and policymakers to consider. First, he advocates for the increased technological specificity of cybersecurity requirements regarding medical devices. Second, to prevent circumvention of the law by putative bad actors, he argues that we ought to apply the so-called ‘bad-man theory’ of law to the case at hand. Third, he suggests that measures to ensure greater privacy are needed. Finally, drawing on the *General Data Protection Regulation*^14^ as an example of good practice, he transposes the idea of ‘privacy by design’ and argues for ‘security by design’; that is, using the law to mandate that cybersecurity principles and values are designed in when it comes to medical devices.

## Regulating Emerging Technological Futures

One of the things that makes medical devices regulation so challenging is the diversity of issues raised by different technological advances, some of which are not envisaged at the time the relevant laws are being drafted. This difficulty is powerfully demonstrated by the final three contributions to the symposium, all of which tackle some aspect or other of emerging medical device technologies.

Catriona McMillan examines the limits of current medical devices regulation when applied to so-called FemTech;^15^ that is, digital tools and devices aimed at women’s health. Specifically, she examines fertility-related FemTech, demonstrating that, depending on how certain apps are marketed, they can avoid being captured by the medical devices regulations. If the explicitly stated purpose of a FemTech app is digital contraception, then, she argues, they fall squarely within the UK’s medical devices regime. However, if they are marketed as ‘merely’ being period trackers, for instance, it is a different story. McMillan cogently argues that, given the risk of unwanted pregnancy and other harms that can befall users of such apps, this is not good enough. She calls for a more stringent approach to software that treads the fine line between being a medical device and a wellbeing app.

Following this, Phoebe Li, Robin Williams, Stephen Gilbert, and Stuart Anderson look at the difficulties of governing AI-enabled medical devices (AIeMD) in their contribution.^16^ As they highlight, the benefit of AI systems is that their performance can be optimised based on repeated cycles of implementation and learning. However, current regulatory and governance regimes are not set up to accommodate such systems, with the evidentiary requirements, in particular, being incompatible with devices whose function may continually optimise and change. With this in mind, they explore the challenges of regulating AI, as highlighted in stakeholder meetings, and explore recent regulatory attempts to meet these challenges, focusing on proposals for a regulatory approach for AIeMD in the UK.

In the final article in this symposium, Antonia Cronin and Rebecca Thom explore the regulatory challenges arising from regenerative medicine technologies such as the bioartificial pancreas.^17^ These novel regenerative medicine technologies incorporate both cellular and non-cellular components, neither of which, they argue, is more important than the other. The hybridity of these novel products in bringing together these components means that current regulations are poorly suited to the case of bioartificial organs. Cronin and Thom illustrate how, in the EU, such products are not adequately covered by the provisions of two key regulations: the EU MDR and the Advanced Therapy Medicinal Products (ATMP) Regulation.^18^ More than that, however, they show how products such as the bioartificial pancreas engage a multiplicity of overlapping regulations and their requirements, making the bringing to market of one of these products highly complex and burdensome. Therefore, they propose that the existing matrix of regulatory oversight for ATMPs should be streamlined to avoid stifling the development of promising technologies.

## Footnotes

1 The “Visions of the Everyday Cyborg” workshop took place at the University of Birmingham in September 2022. It was part of the Wellcome Trust–funded “Everyday Cyborgs 2.0: Law’s Boundary Work and Alternative Legal Futures” project (Grant No: 212507/Z/18/Z).

2 In May 2021 and May 2022, respectively.

3 Proposal for a Regulation of the European Parliament and of the Council on harmonised rules on fair access to and use of data, COM (2022)68 final (“Data Act”).

4 Proposal for a Regulation of the European Parliament and of the Council laying down harmonised rules on Artificial Intelligence and amending certain Union legislative acts, COM(2021)206 final (‘Artificial Intelligence Act’).

5 Regulation (EU) 2019/881 (“Cybersecurity Act”) of the European Parliament and of the Council of 17 April 2019 on ENISA (the European Union Agency for Cybersecurity) and on information and communications technology cybersecurity certification and repealing Regulation (EU) No 526/2013.

6 Directive (EU) 2022/2555 (“NIS2 Directive”) of the European Parliament and of the Council of 14 December 2022 on measures for a high common level of cybersecurity across the Union, amending Regulation (EU) No 910/2014 and Directive (EU) 2018/1972, and repealing Directive (EU) 2016/1148.

7 Quigley, “Medical Devices Regulation.”

8 Medicines and Healthcare products Regulatory Agency, “Consultation on the Future Regulation.”

9 Medicines and Healthcare products Regulatory Agency, “Government Response.”

10 MacLeod, “Independent Medicines and Medical Devices Safety Review”

11 Quigley, “Medical Devices Regulation.”

12 Biasin, “New Cybersecurity Requirements.”

13 Ludvigsen, “Cybersecurity in Medical Devices.”

14 Regulation (EU) 2016/679 of the European Parliament and of the Council of 27 April 2016 on the protection of natural persons with regard to the processing of personal data and on the free movement of such data, and repealing Directive 95/46/EC (General Data Protection Regulation)(“GDPR”).

15 McMillan, “Contraception.”

16 Li, “Regulating Artificial Intelligence.”

17 Cronin, “Regulatory Challenges.”

18 Regulation (EC) No 1394/2007 of the European Parliament and of the Council of 13 November 2007 on advanced therapy medicinal products.
